# Electronic Cigarette Advertising Impacts Adversely on Smoking Behaviour Within a London Student Cohort: A Cross-Sectional Structured Survey

**DOI:** 10.1007/s00408-019-00262-z

**Published:** 2019-08-28

**Authors:** C. Ratneswaran, J. Steier, K. Reed, T. K. Khong

**Affiliations:** 1 Lane Fox Unit/ Sleep Disorders Centre, NHS Foundation Trust, Guy’s and St ThomasWestminster Bridge Road, London, SE1 7EH UK; 2 Faculty of Life Sciences and Medicine, Centre for Human & Applied Physiological Sciences, King’s College London, London, UK; 3 Institute of Medical and Biomedical Education, St George’s, University of London, London, UK

**Keywords:** E-cigarette, Tobacco, Smoking, Public health

## Abstract

**Introduction:**

In contrast to tobacco smoking, electronic cigarette (“vaping”) advertisement had been approved in the United Kingdom (UK) in January 2013. Currently, there are an estimated 3.2 million UK e-cigarette users. The impact of e-cigarette advertisement on tobacco use has not been studied in detail. We hypothesised that e-cigarette advertisement impacts on conventional smoking behaviour.

**Methods:**

A cross-sectional structured survey assessed the impact of e-cigarette advertising on the perceived social acceptability of cigarette and e-cigarette smoking and on using either cigarettes or e-cigarettes (on a scale of 1 to 5/‘not at all’ to ‘a lot’). The survey was administered between January to March 2015 to London university students, before and after viewing 5 UK adverts including a TV commercial.

**Results:**

Data were collected from 106 participants (22 ± 2 years, 66% male), comprising cigarette smokers (32%), non-smokers (54%) and ex-smokers (14%). This included vapers (16%), non-vapers (77%) and ex-vapers (7%). After viewing the adverts, smokers (2.6 ± 1.0 vs. 3.8 ± 1.1, *p* = 0.001) and non-smokers (3.2 ± 0.7 vs. 3.7 ± 0.8, *p* = 0.007) felt smoking was more socially acceptable, compared to before viewing them. Participants were more likely to try both e-cigarettes (1.90 ± 1.03 to 3.09 ± 1.11, *p* < 0.001) and conventional cigarettes (1.73 ± 0.83 to 2.27 ± 1.13, *p* < 0.001) after viewing the adverts compared to before. Vapers were less likely to smoke both an e-cigarette, and a conventional cigarette after viewing the adverts.

**Conclusion:**

E-cigarette advertising encourages both e-cigarette and conventional cigarette use in young smokers and non-smokers. The adverts increase the social acceptability of smoking without regarding the importance of public health campaigns that champion smoking cessation.

**Electronic supplementary material:**

The online version of this article (10.1007/s00408-019-00262-z) contains supplementary material, which is available to authorized users.

## Introduction

The development of e-cigarettes, otherwise known as vapourisers, has led to the delivery of inhaled nicotine without tobacco through a vapourised solution via battery-operated devices [1]. Since their introduction in China in 2004 and in the United States of America in 2007, e-cigarettes have become widely available, and their use has risen exponentially [2]. Currently, there are an estimated 3.2 million adults in Great Britain using e-cigarettes [3]. Despite this figure, many unanswered questions about their safety, efficacy for harm reduction and impact on smoking cessation remain [4, 5]. Further, over a third of current e-cigarette users do so alongside the use of conventional cigarettes [3], putting the overall efficacy of e-cigarettes for smoking cessation into question.

Importantly, e-cigarette advertising remains an area of great controversy. While tobacco products that *do not* claim to have health benefits are subject to licensing by the European Tobacco Products Directive (TPD) [6], in the United Kingdom those that *do* claim to have health benefits are subject to licensing by the Medicine and Health Regulatory Authority (MHRA) [7]. This has important implications as significant differences exist between the two forms of licensing with respect to tobacco products.

Compared to TPD licensing, MHRA regulation leads to lower taxation (5% VAT vs 20% VAT) and no enforcement of package warning labels. It also means that the products are available on prescription and *importantly*, that there is greater flexibility in advertising; this includes cross-border advertising, television commercials and use of billboard and buses [7].

Since the MHRA decided to regulate e-cigarettes as medicinal products, as with any ‘over the counter’ medication, they were granted the right to advertise. In light of this, the Committee on Advertising Practise (CAP) implemented new guidelines for the advertisement of e-cigarettes [8].

In August 2015, Public Health England released a statement to the media suggesting that e-cigarettes are 95% less harmful than conventional cigarettes [9], but there remains a controversy as this statement was met with scepticism from international high-impact factor journals [10, 11] questioning the validity and strength of the data they had cited.

Independent of any potential harm of e-cigarettes, this study focused on the perception and response to e-cigarette adverts. We hypothesised that e-cigarette advertising may encourage the use of e-cigarettes and conventional cigarettes in smokers and non-smokers.

## Methods

The study was approved by the local university research ethics committee, and was performed at Kingston University (KU) and St George’s, University of London (SGUL), during a three-week period from 2nd March to the 20th March 2015. Written informed consent was obtained prior to participation. Inclusion criteria were fluent English, student, age 18–80 years and both genders. Those who were unable to communicate, understand or view the questionnaire or participant information sheets were excluded.

### Structured Survey

A 17-item structured survey was designed following an internal peer-review process from four academic institutions (Guys and St Thomas’ NHS Foundation Trust, King’s College London and St George’s, University of London and Kingston University). A university student population was used as university students/young adults, who are at a unique stage of experimentation and peer influence [12], are at particular risk of smoking initiation [13].

The survey assessed demographics to assess for baseline confounders, smoking risk awareness and perceptions on whether “e-cigarettes are an effective means of helping people to stop smoking tobacco cigarettes” (on a scale of 1/“strongly disagree” to 5/“strongly agree”).

Awareness of the following smoking-related health risks were assessed in each participant to determine whether they influenced the intention to smoke a conventional cigarette after viewing the advertising:

Early menopause [14], fertility [15], ageing [16], rheumatoid arthritis [17], sexual dysfunction [18], chronic obstructive pulmonary disease (COPD) [19], lung cancer [20], blindness [21], strokes [22] and heart disease [23].

Participant’s intention to smoke a conventional cigarette, as well as an e-cigarette was elicited (“how much do you want to try a cigarette/e-cigarette”, on a scale of 1/“not at all” to 5/“a lot”) prior to, and following, viewing five different UK e-cigarette adverts. Participants were further asked how they would prefer e-cigarettes to be regulated.

A structured survey was chosen as the assessment tool, as this was deemed to be the most efficient way to collate the above information, and to evaluate the instant psychological influence of e-cigarette advertising on the intention to use cigarettes or e-cigarettes. Although the dimension of “intention” is thought to have a low correlation with the dimension “action”, “intention” measurements are widely used in this context [24–26] and “intention to smoke” is considered an important predictor of smoking behaviour, as it is included in various health and social psychology theories [25, 26].

### Advertisements Shown

Five images of recent e-cigarette advertisements were utilised, each focussing on different messages: ‘a healthier option to smoking’, ‘beating the smoking ban laws’, ‘no need to quit smoking’ (please refer to Online Supplement Fig. E1). These are typical messages found in e-cigarette advertising, as shown in recent content reviews [27–29].

### Sample Size Analysis

We assumed a minimal mean difference in the overall “intention to smoke” a conventional cigarette, after viewing the e-cigarette advertising, to be 0.5 (on a scale of 0 to 4, with the numbers indicating the difference of “intention to smoke” pre and post viewing the advertisements), with a maximum standard deviation of 1.5. To achieve a minimal power of 80% at a 5% significance level (two-sided), the study would require a sample of 73 participants. With a hypothetical wider standard deviation of 1.75, this would require a sample of 99 participants.

### Statistical Analysis

Data were collected using MS excel 2007 (Microsoft Corporation, Seattle/WA, USA) and analysed using SPSS statistics 22 (IBM, New York/NY, USA). Normality was tested using the Shapiro–Wilks test. Categorical data were assessed using the *X*^2^ test and non-categorical data were analysed using paired and unpaired *t* tests, if normally distributed, and the Mann–Whitney test, if non-normally distributed. A *p*-value of < 0.05 was considered as being significant.

## Results

One hundred and six participants (22 ± 2 years, 66% male) completed the study, comprising cigarette smokers (32%), non-smokers (54%) and ex-smokers (14%). This included vapers (16%), non-vapers (77%) and ex-vapers (7%) (Table 1). The vapers (*n* = 17) consisted of 59% non-smokers and 41% ex-smokers. The non-vapers (*n* = 82) comprised 51% non-smokers, 41% smokers and 7% ex-smokers. The smokers (*n* = 34) comprised 100% non-vapers. The non-smokers (*n* = 57) comprised 74% vapers, 18% non-vapers and 9% ex-vapers.

**Table 1:** Participant demographics; including sub-group comparison of non-smokers versus smokers, and non-vapers versus vapers

	All participants (*n* = 106)	Non-smokers (*n* = 57)	Smokers (*n* = 34)	*p*-Value	Non-vapers (*n* = 82)	Vapers (*n* = 17)	*p*-Value
Age (range)	21.7 (1.8)	22.0 (1.9)	21.4 (1.9)	**0.129**	21.5 (1.9)	21.9 (1.6)	**0.379**
Male (n, %)	(66%)	32 (56%)	25 (74%)	**0.097**	54 (66%)	11 (65%)	**0.928**
Female (n, %)	(44%)	25 (44%)	9 (26%)		28 (34%)	6 (35%)	
Asian/A-British	50 (47%)	29 (51%)	17 (50%)	**0.935**	37 (45%)	12 (71%)	**0.056**
Black/B-British	16 (15%)	10 (18%)	2 (6%)	**0.112**	11 (13%)	3 (18%)	**0.649**
Chinese/C-British	10 (9%)	8 (14%)	1 (3%)	**0.086**	8 (10%)	0 (0%)	**0.129**
Mixed	7 (7%)	4 (7%)	1 (3%)	**0.409**	6 (7%)	1 (6%)	**0.834**
White	23 (22%)	6 (11%)	13 (38%)	**0.001**	20 (24%)	1 (6%)	**0.089**

### Perceptions of E-cigarettes Pre and Post Advertising

Overall, after viewing the e-cigarette advertising, participants felt that smoking conventional cigarettes was more socially acceptable (Δ0.82 ± 0.29 95% CI, *p* < 0.001). Further, they felt that e-cigarettes were more efficient (Δ0.14 ± 0.12 95% CI, *p* = 0.020) than alternative nicotine replacement therapies (NRT) at preventing smoking, compared to before viewing the adverts. Overall, after viewing the adverts the perceived safety of e-cigarettes compared to conventional cigarettes declined (Δ-0.13 ± 0.10 95% CI, *p* = 0.014), although e-cigarettes were still viewed as safer than conventional cigarettes (Table 2).

**Table 2:** Perceptions of e-cigarettes pre and post advertising exposure

	All participants (* n* = 106)			
	Before	After	Δ (95% CI)	p
Effective	3.98 (0.82)	3.94 (0.74)	− 0.04 (0.12)	0.528
Efficient	3.65 (0.92)	3.79 (0.82)	0.14 (0.12)	**0.020**
Safer	4.27 (0.74)	4.14 (0.64)	− 0.13 (0.10)	0.140
Harmful	2.09 (0.83)	2.25 (0.95)	0.15 (0.17)	0.105
Normalising	2.93 (0.95)	3.95 (0.95)	0.82 (0.29)	** < 0.001**

Effective = *e-cigarettes are an effective means to help quit smoking*. Efficient = *e-cigarettes are more efficient that nicotine replacement therapy (e.g. patches) at quitting smoking.* Safer = *e-cigarettes are safer to use that conventional cigarettes.* Harmful = *e-cigarettes are harmful to health.* Normalising = *e-cigarettes are increasing the social acceptability of conventional cigarette smoking*

#### Sub-group Analysis: Smokers and Non-smokers

After viewing the advertisements, cigarette smokers (Δ1.18 ± 0.57 95% CI, *p* < 0.001) and non-smokers (Δ0.44 ± 0.29 95% CI, *p* = 0.007) felt that conventional cigarette smoking was more socially acceptable. Non-smokers, but not smokers, rating of the relative safety of e-cigarettes in comparison to conventional cigarettes declined (Δ− 0.14 ± 0.14 95%CI, *p* = 0.044). In the smoking and non-smoking groups, after viewing the adverts, there were no significant changes regarding the effectiveness of e-cigarettes in smoking cessation, the efficiency of e-cigarettes compared to NRT, or their potential harm to health (Table 3).

**Table 3:** Perceptions of e-cigarettes pre and post advertising exposure: smokers and non-smokers

Total (*n* = 89)	Smokers (*n* = 33)				Non-smokers (*n* = 56)			
	Before	After	Δ (95% CI)	*p*	Before	After	Δ (95% CI)	*p*
Effective	4.21 (0.84)	4.18 (0.72)	− 0.03 (0.28)	0.830	3.74 (0.74)	3.67 (0.61)	− 0.07 (0.14)	0.322
Efficient	3.94 (0.92)	4.09 (0.79)	0.15 (0.21)	0.166	3.28 (0.77)	3.44 (0.68)	0.16 (0.17)	0.072
Safer	4.50 (0.75)	4.32 (0.68)	− 0.18 (0.22)	0.124	4.07 (0.70)	3.93 (0.49)	− 0.14 (0.14)	**0.044**
Harmful	1.94 (0.85)	2.21 (1.07)	0.26 (0.34)	0.150	2.28 (0.77)	2.33 (0.83)	0.05 (0.18)	0.568
Normalising	2.62 (1.04)	3.79 (1.07)	1.18 (0.57)	**0.001**	3.23 (0.68)	3.67 (0.83)	0.44 (0.29)	**0.007**

Effective = *e-cigarettes are an effective means to help quit smoking*. Efficient = *e-cigarettes are more efficient that nicotine replacement therapy (e.g. patches) at quitting smoking.* Safer = *e-cigarettes are safer to use that conventional cigarettes.* Harmful = *e-cigarettes are harmful to health.* Normalising = *e-cigarettes are increasing the social acceptability of conventional cigarette smoking*

#### Sub-group Analysis: Vapers and Non-Vapers

After viewing the advertisements, non-vapers (but not vapers) felt that conventional cigarette smoking was more socially acceptable (Δ0.89 ± 0.34 95% CI, *p* < 0.001). Non-vapers felt that e-cigarettes were more efficient than NRT (Δ0.18 ± 0.14 95% CI, *p* = 0.013) in preventing smoking. Non-vapers rating of the relative safety of e-cigarettes compared to conventional cigarettes also declined (Δ− 0.18 ± 0.13 95% CI, *p* = 0.006). The adverts had no significant impact on the perception of e-cigarettes amongst our current vapers (Table 4).

**Table 4:** Perceptions of e-cigarettes pre and post advertising exposure: vapers and non-vapers

	Vapers (*n* = 16)				Non-vapers (*n* = 81)			
	Before	After	Δ (95% )	*p*	Before	After	Δ (95% CI)	*p*
Effective	3.88 (0.70)	3.82 (0.81)	− 0.06 (0.29)	0.655	4.00 (0.85)	3.95 (0.72)	− 0.05 (0.14)	0.495
Efficient	3.71 (0.69)	3.71 (0.69)	0.00 (0.26)	1.000	3.61 (0.98)	3.79 (0.86)	0.18 (0.14)	**0.013**
Safer	4.12 (0.60)	4.06 (0.66)	− 0.06 (0.12)	0.317	4.33 (0.75)	4.15 (0.63)	− 0.18 (0.13)	**0.006**
Harmful	2.35 (1.00)	2.47 (1.07)	0.12 (0.65)	0.942	2.05 (0.80)	2.23 (0.93)	0.18 (0.18)	0.050
Normalising	3.18 (0.81)	3.53 (1.07)	0.35 (0.68)	0.323	2.88 (0.99)	3.77 (0.93)	0.89 (0.34)	** < 0.001**

Effective = *e-cigarettes are an effective means to help quit smoking*. Efficient = *e-cigarettes are more efficient that nicotine replacement therapy (e.g. patches) at quitting smoking.* Safer = *e-cigarettes are safer to use that conventional cigarettes.* Harmful = *e-cigarettes are harmful to health.* Normalising = *e-cigarettes are increasing the social acceptability of conventional cigarette smoking*

### Impact of Advertisements on Intention to Smoke

Overall, participants were more likely to smoke a conventional cigarette (Δ0.55 ± 1.19 95% CI, *p* < 0.001) and an e-cigarette (Δ1.20 ± 0.26 95% CI, *p* < 0.001) after viewing the e-cigarette advertisements compared to before. Though sub-group analyses revealed that smokers, non-smokers and non-vapers to be more inclined to do so, vapers were actually less likely to smoke both a conventional, and an e-cigarette after viewing the advertising (Table 5).

**Table 5:** Intention to quit smoking, pre and post e-cigarette advertising exposure

	Before	After	Δ (95% CI)	*p*
Intention to conventionally smoke				
OVERALL	1.73 (0.83)	2.27 (1.13)	0.55 (1.19)	** < 0.001**
Vaper	3.00 (0.79)	2.76 (1.15)	− 0.24 (0.59)	**0.009**
Non-vaper	1.77 (0.86)	2.38 (1.38)	0.61 (0.23)	** < 0.001**
Smoker	2.59 (0.61)	3.41 (0.96)	0.82 (0.33)	** < 0.001**
Non-smoker	1.19 (0.44)	1.46 (0.89)	0.26 (0.26)	** < 0.001**
Intention to vape				
OVERALL	1.90 (1.03)	3.09 (1.11)	1.20 (0.26)	** < 0.001**
Vaper	3.00 (0.79)	2.76 (1.15)	− 0.24 (0.59)	**0.009**
Non-vaper	1.59 (0.86)	3.13 (1.12)	1.55 (0.25)	** < 0.001**
Smoker	2.03 (1.06)	3.56 (0.99)	1.53 (0.43)	** < 0.001**
Non-smoker	1.65 (0.95)	2.61 (0.98)	0.96 (0.37)	** < 0.001**
Intention to quit smoking				
OVERALL	1.61 (0.88)	1.29 (0.65)	− 0.32 (0.12)	** < 0.001**
Vaper	2.12 (1.11)	1.53 (0.87)	− 0.59 (0.55)	**0.041**
Non-vaper	1.52 (0.80)	1.26 (0.60)	− 0.27 (0.11)	** < 0.001**
Smoker	1.74 (0.79)	1.38 (0.65)	− 0.35 (0.19)	**0.001**

Participants overall had less intention to quit smoking after viewing the advertisements (Δ− 0.59 ± 0.55 95% CI, *p* = 0.041), compared to before. Sub-group analyses revealed this to also be the case for vapers, non-vapers and smokers (Table 5).

### Knowledge of Smoking-Related Health Consequences

Non-smokers revealed an increased awareness of diseases/conditions related to smoking compared to smokers (for more detailed information please refer to the online Supplementary file Table E2). There was a statistically significant difference (*p* < 0.001) regarding the awareness of smoking-related menopause, rheumatoid arthritis, ageing, blindness (all *p* < 0.001), and also with respect to knowledge of COPD and fertility problems (*p* < 0.05). Current e-cigarette users were more aware of the condition/diseases related to smoking than the non-e-cigarette users (for more detailed information please refer to the online supplementary file, table E3).

## Discussion

E-cigarette advertising increases the social acceptability, and intention to smoke conventional cigarettes in a young student population.

Since the 1970s, tobacco companies have not been able to advertise their products on British television or radio [30]. It is therefore important to understand that e-cigarette advertising potentially markets not only ‘vaping’ but also conventional smoking in a sublime way to new generations.

Despite the advertising standards set out by CAP, e-cigarette adverts are designed to be attractive to all potential users, smokers and non-smokers, of any age. The tobacco industry has an established interest in attracting young people to the market [31]. Media reports suggest that companies producing e-cigarettes are employing similar marketing tactics that were previously used to attract younger people to smoking, by presenting the use of e-cigarettes as a desirable pursuit [27].

While e-cigarettes are marketed as a healthy substitute to conventional smoking, healthy substitutes are known to reinforce the use of the original unhealthy product [32]. E-cigarettes are designed to emulate conventional cigarettes in the way they look and feel and by the inclusion of other characteristic features of conventional cigarettes such as smoke-like vapours and glowing tips during inhalation [33], an effect described in nature as mimicry. However, the similarity to an unhealthier product is reflected in the results of this study where smokers and non-smokers were more likely to try e-cigarettes as well as conventional cigarettes following exposure to adverts.

Overall, viewing the advertisements swayed the rating of the efficiency of e-cigarettes compared to NRT in stopping smoking, in favour of e-cigarettes; this trend was largely driven by non-vapers. Indeed, there is evidence from randomised trials demonstrating the greater effectiveness of e-cigarettes over NRT [34, 35].

The advertisement also made the overall rating of the safety of e-cigarettes in comparison to conventional cigarettes decline, and this was driven by the beliefs of non-smokers and non-vapers. Therefore, it is interesting that despite a decrease in the perception of safety in non-smokers and non-vapers, e-cigarettes advertising still enhanced the impulse to both vape and smoke in all groups apart from vapers, where this appeared to reduce intention to both smoke and vape. This demonstrates the illusory impact electronic cigarette advertising has on overall positive beliefs about e-cigarettes while still targeting the impulse to smoke through their imagery, regardless of concerns about safety.

Notwithstanding CAP regulations, tobacco companies are still presenting e-cigarettes as healthier, safer alternatives to conventional cigarettes [36]. Further, they are advertised as a smoking cessation method. As smoking cessation is considered to be one of the main reasons for e-cigarette use [37], it is thought that advertising should be reinforcing this message. Our study found that most participants were ‘not at all’ convinced that the advertisements made them think about quitting smoking.

The change in attitude favouring an unhealthy behaviour after viewing an advertisement is not unexpected [38], and although the correlation between intended and actual behaviour is known to be weak [39], cigarette advertising in the past has had a harmful impact on society [40]. Interestingly, all participants felt that the adverts were normalising the social acceptability of smoking. This highlights the subconscious impact that advertising has on an individual [41].

### Limitations of this Study

E-cigarette users came from a university, which involved public health or science (e.g. pharmacy, sport science, nursing) and this may be a confounder of the data. The study was conducted on a specific cohort (age group) in a single centre and the results may not be generalisable to a whole population and should be interpreted with caution. Any investigator led bias was eliminated as only one investigator collated data.

In addition, there might be a selection bias towards those that were willing to participate. Students who were already well versed around e-cigarettes, or whom had already had formulated views, may have been more likely to take part, skewing our results. With this in mind, the introduction to the study was kept neutral in order to obtain data from participants independent of the view of electronic cigarettes.

Several e-cigarette adverts were shown to participants within a short time period and this could have caused a greater emotional response than showing single advertisement images over a longer time. This might further lead to a negative response when viewing them. Future study designs should aim to study the longitudinal effect of exposure to adverts to allow for a similar effect as in real life.

Our survey was designed by internal peer review through four academic institutions and based on three previously published studies [42–44]. Our primary outcome of intention smoke a conventional cigarette was assessed with a likert scale ranging from 1 to 5. We included this single question to reduce the survey burden on the participant and ensure accurate completion, though this method to determine intention to smoke has not been assessed for validity or reliability. We had a 100% survey completion rate suggesting that survey fatigue was not an issue. Future studies should include validated and reliable measures for intention to smoke such as the three-item scale by Pearson et al. [45].

Further, studies could also utilise conditional risk assessments, where participants are asked to consider potential outcomes if they, hypothetically, were to engage in that particular behaviour (e.g. “what is the chance you would become unwell if you were to smoke a conventional cigarette”). Such measures have shown to be a superior indicator of behaviour [46], and also have the added benefits of revealing the underlying beliefs which may drive that behaviour.

Lastly, while we have classified participants into smokers, non-smokers and ex-smokers; however, no distinction was made between different smokers in terms of the number of cigarettes per day, or the duration they had been smoking. These are important factors to be determined in future studies, as it is known that university students, who are young and may have a shorter smoking history, may predominantly be light or intermittent smokers [47]. Light and intermittent smokers are believed to be less susceptible to tobacco prevention messages, as they do not consider themselves being typical smokers [48, 49], although these cohorts are at risk of developing long-term smoking habits with reduced concerns about smoking-related consequences [50, 51]. It is therefore important to understand how e-cigarette advertising may impact the propensity to develop consolidated or worsening smoking habits in various groups of smokers.

## Conclusion

Electronic cigarette advertisement has a negative impact on the smoking perception amongst students. The advertisements influence young people, irrespective of their previous smoking habits, towards smoking e-cigarettes and conventional cigarettes. It also impacts on their acceptance of smoking behaviour and fails to encourage smoking cessation. With the advent of e-cigarettes advertisement, public health faces a new marketing campaign that promotes tobacco industry products that mimic smoking. As the popularity of e-cigarettes continues to thrive, not enough is being done to explore their safety and efficacy or the marketing tactics being used to sell them.

## Electronic supplementary material

Below is the link to the electronic supplementary material.

Supplementary file1 (DOCX 1837 kb)
