# Supplementary material for: Electronic Cigarette Advertising Impacts Adversely on Smoking Behaviour Within a London Student Cohort: A Cross-Sectional Structured Survey
Source: Lung. 2019 Aug 28;197(5):533–40. doi: 10.1007/s00408-019-00262-z (PMC6778585; doi:10.1007/s00408-019-00262-z)
Supplement: Supplementary file 1 — Supplementary file1 (DOCX 1837 kb) [file 408_2019_262_MOESM1_ESM.docx]

**Electronic cigarette advertising impacts adversely on smoking behaviour within a London student cohort: a cross-sectional structured survey**

Online Supplementary File

Ratneswaran C^1,2,3^, Steier J^2,3^, Reed K^3^, Khong TK^1^

Figure E1: E-cigarette advertising used within the study, which were currently being used at the time. References are included under the advert, if not within them.


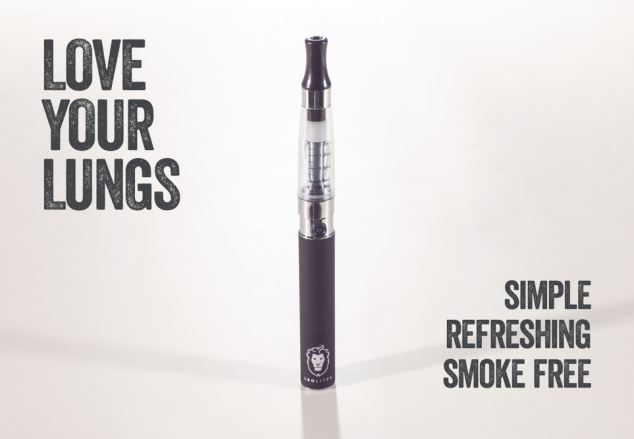

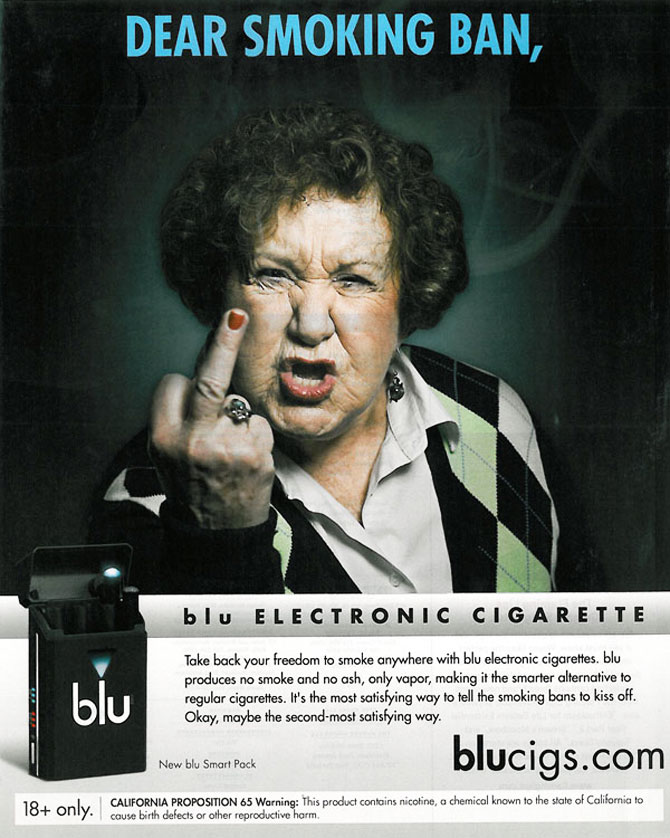


[Leolite.com](http://www.lLeolite.com)

LeoLite e-cigarette manufacturers


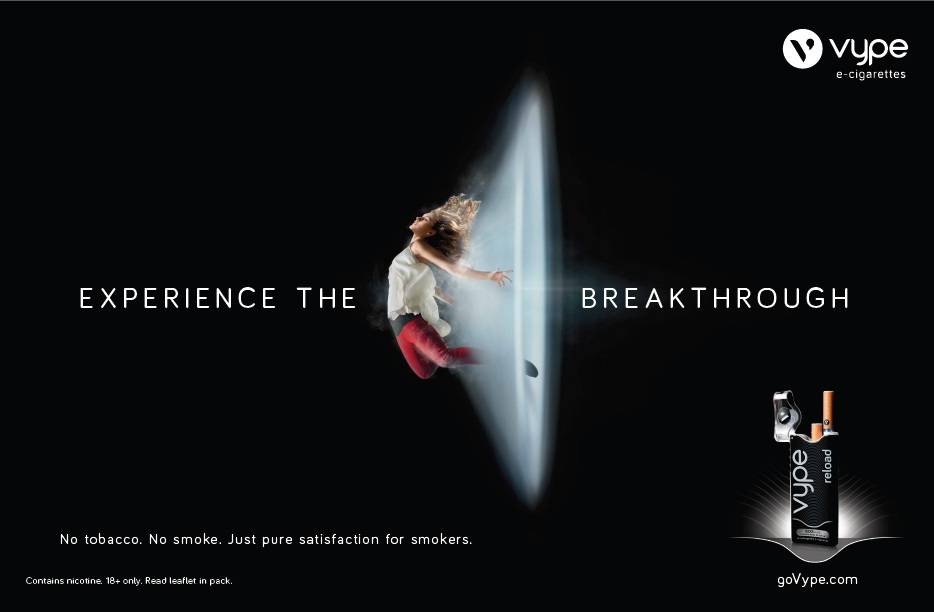


[Govype.com](http://www.lLeolite.com)

Vype e-cigarette


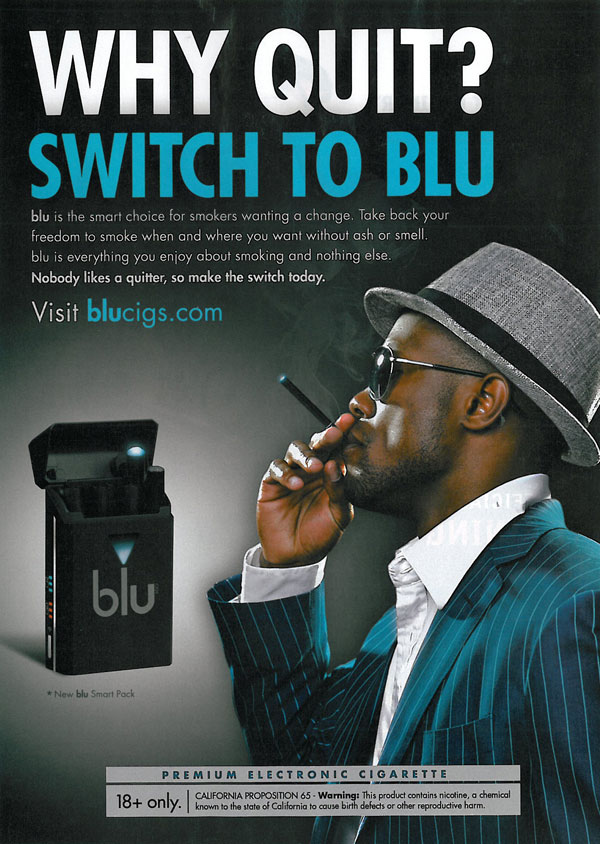


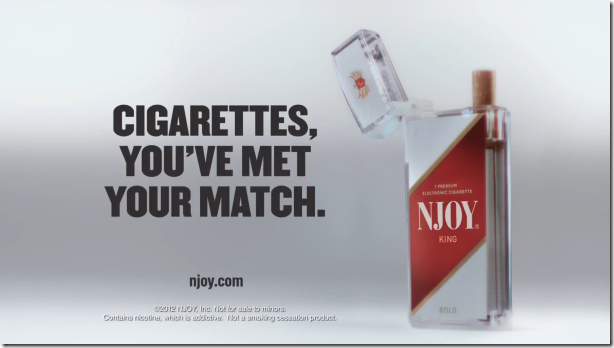


[Njoy.com](http://www.lLeolite.com)

E-cigarette manufacturers

Blucigs.com

E-cigarette manufacturers

Figure E2: Awareness of smoking associated consequences: Smokers *vs* non-smokers (%yes)

Figure E3: Awareness of smoking associated consequences: Vapers *vs* non-vapers (%yes)
